# *BRCA* mutation rate and characteristics of prostate tumor in breast and ovarian cancer families: analysis of 6,591 Italian pedigrees

**DOI:** 10.20892/j.issn.2095-3941.2020.0481

**Published:** 2021-06-15

**Authors:** Laura Cortesi, Federica Domati, Annalisa Guida, Isabella Marchi, Angela Toss, Elena Barbieri, Luigi Marcheselli, Marta Venturelli, Simonetta Piana, Claudia Cirilli, Massimo Federico

**Affiliations:** 1 Department of Oncology and Hematology, University Hospital of Modena, Modena 41124, Italy; 2 Department of Medical and Surgical Sciences for Children & Adults, Division of Medical Oncology, University Hospital of Modena, Modena 41124, Italy; 3 Pathology Unit, Azienda USL Reggio Emilia, IRCCS, Reggio Emilia 42123, Italy; 4 Modena Cancer Registry, Public Health Department, AUSL Modena 41126, Italy

**Keywords:** *BRCA* genes, prostate cancer, hereditary cancer, Modena criteria, breast cancer, ovarian cancer

## Abstract

**Objective:**

As prostate cancer (PrC) shows a *BRCA* mutation rate as high as 30%, it becomes crucial to find the optimal selection criteria for genetic testing. The primary objective of this study was to evaluate the *BRCA* mutation rate in families with PrC associated with breast and/or ovarian cancers; secondary aims were to compare the characteristics of families and BRCA-related PrC outcome among *BRCA1* and *BRCA2* carriers.

**Methods:**

Following the Modena criteria for the *BRCA* test, we evaluated the mutation rate in families with breast and/or ovarian cancer with a Gleason score ≥7 PrCs, by testing breast or ovarian cases and inferring the mutation in the prostate cases. The characteristics of families and BRCA-related PrC outcomes were measured using the chi-square (χ^2^) test and Kaplan–Meier methods, respectively.

**Results:**

Among 6,591 families, 580 (8.8%) with a Gleason score ≥ 7 PrCs were identified, of which 332 (57.2%) met the Modena selection criteria for BRCA testing. Overall, 215 breast or ovarian cancer probands (64.8%) were tested, of which 41 resulted positive for *BRCA* and one for *CHEK2* genes (19.5%). No statistically significant differences were found in BRCA-related PrC prognosis and in the characteristics of families among BRCA1, BRCA2 and non-tested patients. Ten of 23 (44%) mutations in the *BRCA2* gene fell in the prostate cancer cluster region (PCCR) at the 3′ terminal of the 7914 codon.

**Conclusions:**

It appears the Modena criteria are very useful for BRCA testing selection in families with breast and/or ovarian cancer and PrC. A trend toward a worse prognosis has been found in *BRCA2* carriers.

## Introduction

Prostate cancer (PrC) is the most common malignancy among males representing 7.1% of all cancers and a mortality rate of 3.8% in men^1^. In Italy the 5-year survival rate for men with PrC is 92% and the 10-year survival rate is 90%^2^. The etiology of PrC has been the subject of numerous studies and remains largely unknown compared to other common cancers. The well-established PrC risk factors are advanced age, ethnicity, genetic factors, and family history^3^. Other factors associated with PrC include diet (increased consumption of saturated animal fat and red meat, lower intake of fruit, vegetables, vitamins, and coffee), obesity and physical inactivity, inflammation, hyperglycemia, infections, and environmental exposure to chemicals or ionizing radiation. However, an increasing number of elderly men are being diagnosed with PrC due to increased life expectancy and increased prostate specific antigen (PSA) screening. It has also been observed that the risk of PrC mainly increases in Caucasian males over 50 years of age who have no PrC family history, and in Black males over 40 years of age or men with a familial history of PrC^4^. The disease appears to be linked to hereditary factors in a minority of cases (≤ 30%)^5^. *BRCA1* and *BRCA2* genes in prostate tumorigenesis have been associated with more aggressive disease and poor clinical outcomes. The estimated lifetime risk is 9.5% for *BRCA1* and 20% for *BRCA2* mutation carriers^6^. Overall, *BRCA1/2* mutation carriers present an increased risk for breast cancer (BC) (52%–72% in BRCA1, 45%–84% in *BRCA2*), ovarian cancer (OC) (39%–63% in BRCA1, 11%–27% in BRCA2), PrC (3.4-fold increased risk in BRCA1, 8.6-fold increased risk in BRCA2), and pancreatic cancer (PC) (1%–3% in BRCA1, 2%–7% in BRCA2)^7–11^. The identification of a mutation in *BRCA* genes plays a crucial role in the management of hereditary cancer prevention, diagnosis, and treatment. According to the guidelines of the Italian Association of Medical Oncology (AIOM), which reflect the Modena criteria^12–14^ listed in **Table 1**, the Italian National Health Service provides free BRCA diagnostic tests exclusively for breast, ovarian and pancreatic cancer patients, and to healthy individuals with an estimated risk of carrying a *BRCA* mutation ≥ 40%. This large, single-institution cohort study sought to firstly evaluate the rate of BRCA-positive families among those presenting a family history of PrC associated with BC and/or OC selected by Modena criteria and secondly, to explore family history and outcomes of BRCA-related PrCs.

**Table 1:** The Modena Criteria (AIOM Guidelines 2019)

BC and OC diagnosed in the same patient.
OC, fallopian tube, or primary peritoneal cancer (excluded mucinous and borderline) at any age.
Male BC.
Triple negative BC diagnosed ≤ 60 years.
BC diagnosed ≤ 35 years.
At least one BC and at least one OC.
At least two first-degree blood relatives with BC, at least one diagnosed ≤ 40 years or bilateral.
Healthy individuals with an estimated risk of carrying a *BRCA* mutation ≥ 40%, calculated with the BRCAPro risk calculator (Version CaGene6)

BC, breast cancer; OC, ovarian cancer.

## Materials and methods

### Study population and design

The human investigations were reviewed and approved by the Modena Human Investigations Committee (Approval No. 209/16/3387). The patients provided their written informed consent to participate in this study.

Patients with a family history of BC and/or OC who received genetic counseling for risk category classification and the *BRCA* test at the Modena Family Cancer Clinic (MFCC) from 1991, were eligible for the purpose of this analysis. Probands affected with BC or OC who met the Modena criteria for genetic testing could undergo the BRCA analysis and in the case of a positive result, could ease cascade analysis thus favoring access to risk-reducing surgeries, chemo-preventive studies, or more intensive surveillance programs. Family history that included PrC with Gleason score ≥ 7 in a first- or second-degree relative (in the case of female interposition) of BC or OC, were evaluated for this analysis.

A retrospective medical record review of the pedigrees with a Gleason score ≥ 7 PrCs, previously collected during genetic counseling visits, was completed for the present study.

### BRCA testing procedures

Before 2014, genetic testing of *BRCA1* and *BRCA2* genes at our institution was carried out by direct Sanger sequencing, whilst after 2014, genetic testing was performed using next generation sequencing (NGS). The NGS workflow benefits from the use of the Ion AmpliSeq TM (Thermo Fisher Scientific, Waltham, MA, USA) technology that was handled initially with a semi-automated and subsequently, with a fully automated procedure for multiplex polymerase chain reaction (PCR)-based library preparation sequencing on the Ion Torrent platforms (Thermo Fisher Scientific, Waltham, MA, USA). More recently a Multigene Panel Testing provided by MySeq Dx System (Illumina, San Diego, CA, USA), comprising 27 hereditary syndrome genes, was used. Sanger sequencing was routinely performed to validate candidate mutations, as long as multiplex ligation probe amplification (MLPA, MRC-Holland, Amsterdam, The Netherlands) was carried out on a blood sample to detect copy number variations in all genes, except *BARD1*, *BRIP1*, and *NBN*. Sequence alignment, base calling, variant filtering, and annotation process took advantage of the Torrent Software Suite (Thermo Scientific) and of a custom designed bio-informatic pipeline, as described in previously published works^15,16^. Pathogenicity was defined as categories 4 (likely pathogenic) and 5 (pathogenic) of the ENIGMA classification^17^. Classes C3 (unclassified variants), C2 (probably benign), or C1 (benign) were not reported.

### Statistical analysis and outcome measures

A database was set up at our health center, which consisted in collecting family and individual information, surveillance and follow-up data, additional investigation, and the outcome of all examinations. The reports of these families included test results (if performed), age at cancer onset, histology, relation to the proband, vital status, and type of other cancers. We calculated the median follow-up of the proband in the period from 1991 to 2019. The PrC follow-up was calculated from the date of cancer onset to the endpoint of interest: the date of death or the end of the study period. The chi-square (χ^2^) test was used to determine differences in clinicopathological features between groups. All statistical tests were two-sided. Survival curves were estimated using the Kaplan–Meier method including the log-rank test group comparison.

## Results

### *BRCA* mutation rate

**Figure 1:**
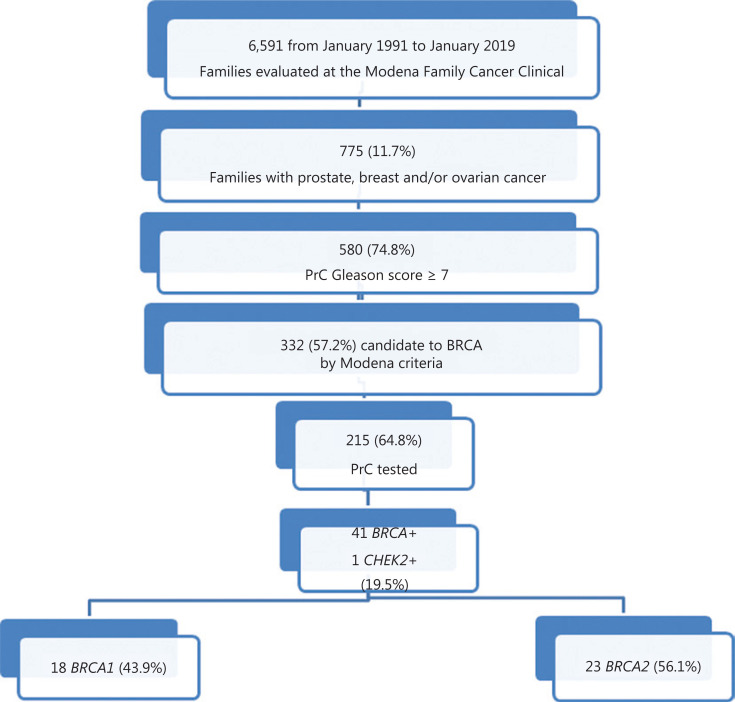
The flow-chart of families evaluated and tested at the MFCC.

From March 1991 to August 2019, 6,591 families with history of BC and/or OC, were counseled and used as the reference population by the MFCC. Seven hundred and seventy-five (11.7%) families reported a family history of associated PrC. Among those PrCs, 580 (74.8%) had a Gleason score ≥ 7. According to the 2019 Modena criteria, 332 (57.2%) of these families were eligible for BRCA testing. In total, 215 probands affected with BC or OC were tested (64.8%), mostly with BC (203) and 12 with OC. The remaining patients were not tested because they refused to or were unable to undergo genetic analysis due to poor health conditions or because patients were not in close proximity to the medical center. Forty-one families were found to be *BRCA* mutation carriers and one family showed a *CHEK2* mutation defined c.1100delC, typically associated with PrC (19.5%). Among the *BRCA* mutated patients, 18 were *BRCA1* positive (43.9%) and 23 were *BRCA2* mutated families (56.1%). We inferred that *BRCA* mutations were also found in PrCs, without testing any prostate cancer case. The diagram of patient selection is represented in **Figure 1**.

### Family characteristics and patient outcomes

The mean age of PrC onset in all population was 68.3 years (range 21–90 years of age), with the lowest age in the *BRCA2* carriers (63.2 years) compared with *BRCA1* carriers (67.9 years) (*P* = 0.84). In the BRCA1 and BRCA2 groups, four (22.2%) and nine (39.1%) families showed two or more PrC cases, respectively, compared with 99 (18.4%) multiple PrCs found in other families (*P* = 0.195). In **Table 2** the characteristics of mutated BRCA and not families are described. The stomach and melanoma tumor rates were increased in *BRCA2* mutation carriers *vs BRCA1* carriers (*P* = 0.136 and *P* = 0.347, respectively). However, no statistically significant differences were seen among all three populations with regard to other associated tumors. The only *CHEK2* mutation shown was described in a family with PrC, BC and bladder cancer.

**Table 2:** Characteristics of family history

Variable	*BRCA1*+ N° 18	*BRCA2*+ N° 23	All study population	P*
Mean age (years)	67.9 (50–87)	63.2 (45–78)	68.3 (21–90)	0.840
Other tumors in family				
Multiple prostate cancer (PrC)	4 (22.2%)	9 (36.3%)	99 (18.4%)	0.195
Colorectal cancer	4 (22.2%)	4 (18.2%)	124 (23.1%)	1.00
Stomach cancer	1 (4%)	6 (27.2%)	75 (14%)	0.136
Melanoma	1 (4%)	2 (8.7%)	19 (3.5%)	0.347
Pancreatic cancer	2 (11%)	2 (9.1%)	4 (6.9%)	0.878
Gallbladder cancer	0	1 (4.5%)	0	0

*P < 0.05 was considered statistically significant.

**Figure 2:**
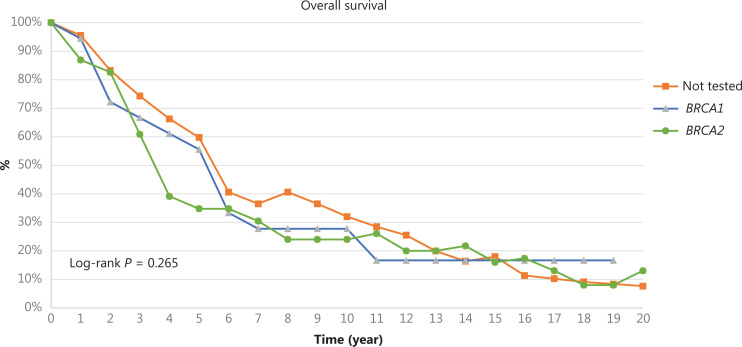
Overall Survival of PrC at 240 months of median follow-up. The log-rank test between non-tested (orange line) and *BRCA1* mutation carriers (blue line) is equal to *P* = 0.911; the log-rank test between non-tested (orange line) *vs BRCA2* mutation carriers (green line) is equal to *P* = 0.265; the log-rank test between *BRCA1* mutation carriers (blue line) and *BRCA2* mutation carriers (green line) is equal to *P* = 0.575.

After 240 months of follow-up, the median overall survival of PrC, evaluated from diagnosis to date of death or last follow-up, was equal to 65 months in a non-tested group of patients, 62 months in patients with BRCA1-related PrC and 40 months in PrC-related BRCA2 patients (*P* = 0.265) (**Figure 2**).

### *BRCA* pathogenic variants and prostate cancer risk

**Figure 3:**
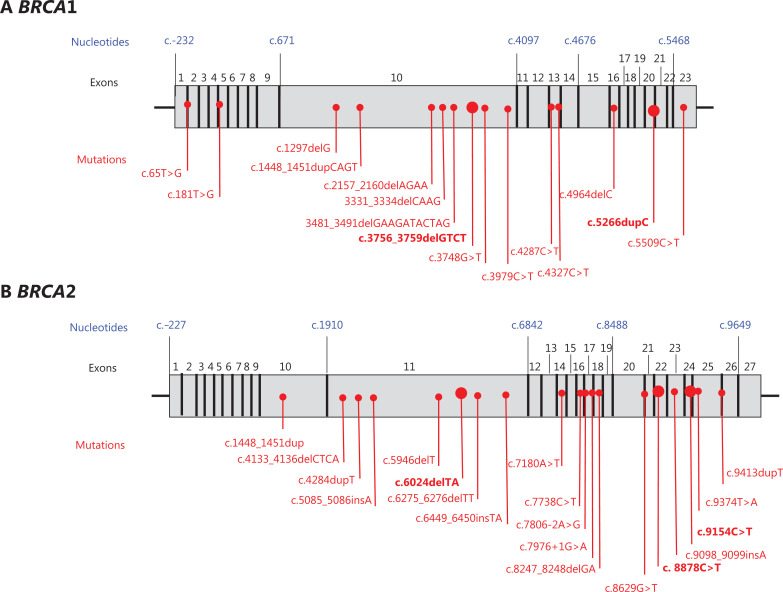
Distribution of mutations along *BRCA1* (A) and *BRCA2* (B) genes. Mutations detected more than once are represented in bold characters.

*BRCA* mutations detected in families with history of PrC are reported in **Figure 3A** and **Figure 3B**. Eleven of 18 (61%) *BRCA1* mutations were located on exon 10. On the other hand, 10 of 23 (44%) mutations in the *BRCA2* gene fell in the prostate cancer cluster region (PCCR) at the 3′ terminal of the 7914 codon. Finally, the most common *BRCA1* mutation types were frame-shift mutations (11 of 18, 61%), followed by missense mutations (7 of 18, 39%). In the *BRCA2* gene, the most frequent mutations were similarly frame-shift mutations (12 of 23, 52%).

## Discussion

Approximately 7%–8% of all patients with PrC has an underlying gene defect on the *BRCA1*, *BRCA2*, and *CHEK2* genes^5^. Furthermore, a recent population study performed on Scandinavian twins, shows a very high rate of hereditary PrC conditions, reaching a high of 57%^18^. Particularly, the *HOXB13* gene has been associated with early onset of PrC in 6% of cases^19^. Moreover, data on positive family history for PrC show a high rate of first-degree relationship for this type of cancer (32.3%)^20^. It therefore becomes crucial to find the optimal selection criteria for genetic testing, with a positive ratio between cost and effectiveness, in order to ease cascade analysis of at-risk relatives and offer effective surveillance programs, based on annual PSA and prostatic ultrasound, that have recently demonstrated to increase the detection of early stage prostate tumors, particularly in case of *BRCA2* mutation carriers^21^.

Patients selected according to the Modena criteria reached a 19.5% of mutations considering all the families eligible for the test, overcoming the probability rate of 10% to find a heterozygous mutation that justifies BRCA screening, as suggested in many countries in accordance with National Institute for Health and Care Excellence (NICE) guidelines^22^. Assuming that first-degree or second-degree (in case of female interposition) relatives with high grade PrC carry the *BRCA* mutation, it can be concluded that the Modena criteria should be introduced in clinical practice for PrC genetic testing. Furthermore, we think that the recent introduction of the multigene panel test could increase the rate of hereditary PrC by adding new genes to the BRCA analysis such as CHEK2, that is frequently associated with this kind of tumor, as was shown recently^23^.

Although no statistically significant differences were seen among BRCA-positive and non-tested families, a trend toward an increased number of PrC and stomach cancer cases was shown in *BRCA2* mutated families. These data are consistent with other previously published studies^24,25^.

Finally, our survival data seem to reflect a worse prognosis of *BRCA2* carriers in comparison with BRCA1 and non-tested patients, although no statistically significant differences have been found. These results can be compared with data from Narod et al.^26^ where the median survival for BRCA2 PrC was 4 years, *vs* 8 years in BRCA1 patients.

Of particular concern, 44% of *BRCA2* mutations were found in the PCCR region at the 3′ terminal of the 7914 codon, recently identified by Patel et al.^27^. It could therefore be useful to identify these mutations, as the risk of PrC in men carriers is high thus inducing us to intensify prostate surveillance programs starting at an earlier age and including other imaging diagnostic tools such as multiparametric magnetic resonance imaging (MRI).

Even though there are some limitations to our retrospective analysis it should be noted that in this study, the BRCA test was performed on patients affected by BC or OC and the positive result was transferred on patients affected by PrC along the genealogy in which the hereditary transmission was found. As most PrC patients were not alive at the time of genetic counseling, it was not possible to verify which PrC patients in BRCA positive families were *BRCA* carriers.

Therefore, a bias may have occurred, as there is a chance, that some cancer cases and their outcome, were not *BRCA* carriers, being phenocopies in *BRCA* mutated families. The high percentage of BRCA1 as compared to BRCA2 is not typical from most PrC series and may reflect biases based on this study population. Furthermore, we are well aware that a bias may have occurred, as only 215 families were tested.

In order to improve our data, we are currently evaluating the possibility to perform a prospective study in which the BRCA test is carried out on PrC tissue thus enabling us to also include deceased patients in future analysis.

In conclusion, our analysis found that Italian criteria are very effective to detect *BRCA1* or *BRCA2* mutations in PrC and should be taken into consideration when deciding upon testing. Data on family history and PrC outcomes in *BRCA2* mutation carriers show an increased number of multiple PrC and stomach cancers and a worse prognosis, as compared with previously published results. Finally, as 44% of *BRCA2* mutations were found in the PCCR region at the 3′ terminal of the 7914 codon, we believe such a variant in healthy men could be useful to intensify a prostate surveillance program at earlier age including the use of multiparametric MRI.
